# Steatotic liver disease in people with HIV at Tshepong Hospital: A post-mortem analysis

**DOI:** 10.4102/sajhivmed.v25i1.1638

**Published:** 2024-12-20

**Authors:** Aqeela Moosa, Ebrahim Variava, Alistair D. Calver, Gajendra Chita, Nadia Sabet, Sharol Ngwenya, Maria Papathanasopoulos, Tanvier Omar

**Affiliations:** 1 Department of Internal Medicine, Faculty of Health Sciences, University of the Witwatersrand, Klerksdorp, South Africa; 2 Perinatal HIV Research Unit, Faculty of Health Sciences, University of the Witwatersrand, Johannesburg, South Africa; 3 Department of Anatomical Pathology, Faculty of Health Sciences, University of the Witwatersrand, Johannesburg, South Africa; 4 HIV Pathogenesis Research Unit, Faculty of Health Sciences, University of the Witwatersrand, Johannesburg, South Africa; 5 Infectious Diseases and Oncology Research Institute, Faculty of Health Sciences, University of the Witwatersrand, Johannesburg, South Africa

**Keywords:** HIV, steatotic liver disease, steatosis, histology, obesity, MASLD

## Abstract

**Background:**

Liver disease is the leading cause of non-AIDS-related mortality in people living with HIV (PLWH). Steatotic liver disease (SLD) is increasingly recognised as an important aetiological factor in liver dysfunction in PLWH.

**Objectives:**

This study aimed to determine the post-mortem prevalence and severity of SLD and determine HIV- and non-HIV-related risk factors associated with it.

**Method:**

We conducted a retrospective cross-sectional study in which liver histology from 59 deceased people who were infected with HIV was assessed for steatosis, and findings correlated with clinical, epidemiological, and biochemical data.

**Results:**

Decedents were predominantly men (33/59); 63% (37/59) were virologically supressed. Median CD4+ T-cell count was 139 cells/µL (interquartile range [IQR]: 47–344). Steatosis was present in 39% (23/59) of decedents: 74% mild, 9% moderate, and 17% severe steatosis. There were no cases of steatohepatitis, and one case with mild fibrosis. Factors associated with SLD were: CD4 T-lymphocyte count > 200 cells/µL (odds ratio [OR]: 3.69; 95% confidence interval [CI]: 1.19–11.44), female sex (OR: 8.5; 95% CI: 2.57–28.17), hypertension (OR: 6.5; 95% CI: 2.05–21.00), and being normal or overweight (OR: 6.75; 95% CI: 1.12–40.56). Virological suppression and duration of antiretroviral drug use were not associated with steatosis.

**Conclusion:**

We found a high proportion of SLD with heterogeneous causes in deceased people who were infected with HIV, exceeding previously reported prevalences from elsewhere in Africa. A preserved CD4 count and being female conferred the highest risk for steatosis, underscoring the need for screening in this subgroup and further research to delineate risks in a Southern African population.

**What this study adds:** This histology-based study from Africa found prevalence of steatotic liver disease approaching that seen in high-income countries. This may indicate an area where prevention and screening resources may need to be directed as more people are controlled on ART.

## Introduction

The HIV pandemic is an ongoing global health issue, with South Africa being home to the largest population of people living with HIV (PLWH), with over 7.9 million infected people and a national prevalence of 14%.^1^ Antiretroviral therapy (ART) has significantly reduced the incidence of opportunistic infections through immune reconstitution. As PLWH survive longer on ART, there is a significant rise in morbidity and mortality from non-AIDS-related diseases, including acute and chronic liver disease.^2^ The aetiologies of liver pathology in PLWH are multifactorial, frequent causes including hepatitis B and C infection, drug-induced liver injury, and steatotic liver disease (SLD). Causes of SLD are varied, encompassing hepatotoxic viruses, drugs, genetic predisposition, alcohol misuse, and metabolic dysfunction-associated SLD (MASLD), a replacement for the old terminology: non-alcoholic fatty liver disease (NAFLD). MASLD recognises multifactorial contributors to liver injury resulting in steatosis in conjunction with metabolic dysfunction.^3^

### Epidemiology

Using diagnostic criteria that were more exclusionary, the worldwide prevalence of NAFLD in the general population is estimated to be 25%, with Africa having the lowest estimated prevalence, at 13.5%.^4^ Studies from Africa are few with small sample sizes. Using imaging-based diagnostics, the prevalence of SLD in PLWH ranges from 13.3% in Nigeria to 35% in Germany.^5,6^ In the South African context, a retrospective study of liver disease in PLWH found a 28% biopsy-proven prevalence of SLD.^7^

### Pathogenesis of liver injury in HIV

The pathogenesis of SLD in PLWH is complex and multifactorial, thought primarily to be driven by insulin resistance, mitochondrial dysfunction, and concurrent viral infections. Insulin resistance is a consequence of the chronic inflammatory state induced by HIV infection, leading to dysregulation of glucose metabolism and dyslipidaemia.^8^ While the pathogenesis of mitochondrial damage is poorly understood, prolonged use of ART, from several classes including nucleoside reverse transcriptase inhibitors (NRTIs), non-NRTIs, and protease inhibitors, has been implicated in inducing mitochondrial dysfunction seen within the liver.^8^

Some studies suggest that HIV increases the relative risk for the development of type 2 diabetes mellitus,^9^ a significant driver of MASLD, which is becoming more prevalent in sub-Saharan Africa since the introduction of ART.^10^ The recent replacement of efavirenz by dolutegravir in the first-line ART regimen in South Africa may result in a rise in the prevalence of obesity in PLWH, as found in the ADVANCE study, with an associated risk for MASLD.^11^

### Diagnosis

Various imaging modalities are used in clinical practice for the diagnosis of SLD; however, liver biopsy for histological evaluation remains the gold standard for diagnosis of SLD, as it also allows for the detection of fibrosis and steatohepatitis.

The interplay between HIV infection, ART, and metabolic factors in the development of SLD remains understudied. In this study, we aimed to determine the post-mortem prevalence and severity of SLD in a South African cohort of deceased people who were infected with HIV, to ascertain both HIV- and non-HIV-related risk factors associated therewith, and to determine likely aetiologies. We believe that a better understanding of the parameters associated with SLD could direct interventions that aim to reduce morbidity and mortality from liver disease in PLWH.

## Research methods and design

### Study design and participants

This retrospective, cross-sectional study was nested within a larger post-mortem study.^12^ Participants were recruited from Tshepong Hospital in the North-West province of South Africa, servicing a population with a high HIV prevalence of 20%.^13^ Decedents were ≥ 18 years, enrolled in the parent study between 01 May 2018 and 30 June 2022. Researchers from the parent study communicated that causes of death were predominantly related to respiratory failure from pneumonia, sepsis, tuberculosis, gastroenteritis, and acute kidney injury. Minimally invasive tissue sampling (MITS) was undertaken post-mortem to detect HIV viral reservoirs and for histological assessment of cause of death. For this study, archived core liver biopsies from the cause of death study have been histologically assessed for the presence of SLD, and these histological findings have been correlated with a subset of epidemiological and biochemical data, the ante-mortem clinical notes, and the documented consensus diagnoses reached by the parent study. Data on body habitus were extracted from patient files: if a treating clinician described a participant as wasted, cachectic, underweight, or reduced body mass index (BMI), patients were classified as underweight; for classifying decedents as overweight, the file descriptors obese, increased BMI, and increased abdominal circumference were used. If alcohol use was noted in the file, it was recorded as positive. All archived liver biopsies were histologically reviewed using routine haematoxylin and eosin staining for overall morphology, reticulin staining for architectural integrity and collapse, and Masson’s trichrome staining to assess for fibrosis. The review was conducted under the supervision of two senior anatomical pathologists.

All liver cores were examined for the presence of steatosis. Steatosis was diagnosed if > 5% hepatic fat was noted. Steatosis was graded according to the Brunt grading system^14^ as follows: mild, 5% – 33% fat; moderate, 33% – 66% fat; and severe, > 66% fat. We evaluated steatohepatitis using Brunt grading for lobular and portal inflammation, and fibrosis. Each biopsy was assessed for the presence of glycogenated nuclei, commonly seen in diabetics, the presence of lipogranulomas, seen in both alcoholic steatohepatitis (ASH) and metabolic-associated steatohepatitis (MASH), and microgranulomas, sometimes seen in association with drug-induced liver injury. For an alcohol-related aetiology, a search was undertaken for Mallory Denk bodies, ballooning degeneration of hepatocytes, lipogranulomas, and giant mitochondria. We noted any other abnormal histological findings in each sample. To determine the probable cause for steatosis, where present, the clinical history, serological and histological findings, and clinicopathology conference (CPC) findings from the parent study were reviewed for each case. The CPCs were conducted by three senior physicians and a senior histopathologist, who undertook a detailed patient file review combined with post-mortem histology findings to assess cause of death for each case. We used information generated by these CPCs together with the review of liver histology, which looked at patterns of injury and fat deposition, to determine probable cause for steatosis in each case. The following criteria were applied to assess if MASLD was present: hepatic steatosis, not due to another cause or at least equally likely; and at least one of the following: obesity, type 2 diabetes mellitus, and metabolic dysregulation (increased waist circumference, increased blood pressure, elevated triglycerides, reduced high density lipoprotein cholesterol, and pre-diabetes).^3^ A drug-related aetiology for SLD was considered if the histological features were suggestive thereof, and this was coupled with the appropriate drug history.

Inclusion criteria for this study were decedents who underwent MITS and were all HIV positive. We excluded cases where there was an inadequate liver histology sample, cases where consent was withdrawn, and cases where those enrolled were found to be HIV negative.

### Statistical analysis

Descriptive statistics were calculated for all variables using Stata/BE 17 (StataCorp LLC, College Station, Texas, United States) statistical software for analysis. Fischer’s exact and Mann-Whitney U tests were used to test for differences in baseline characteristics. Odds ratios (ORs) for risk factors associated with steatosis were calculated using logistic regression. Statistical significance was set at a *P*-value of < 0.05. For variables with missing data, we postulated these were missing at random, and because they accounted for less than 30% within each parameter, we chose to exclude those cases from the respective analysis. In the assessment of body habitus, sample size for the obese and normal categories were too small to analyse individually and were collapsed into one category.

### Ethical considerations

Ethics clearance for this study was obtained from the University of the Witwatersrand Human Research Ethics Committee (study approval number: M220638). Written consent for MITS was obtained from decedents’ next-of-kin by the parent study, who stored data, devoid of personal identifiers, in a protected data bank. Permission to use their data bank and histology samples was obtained from the parent study.

## Results

### Clinical characteristics

**FIGURE 1:**
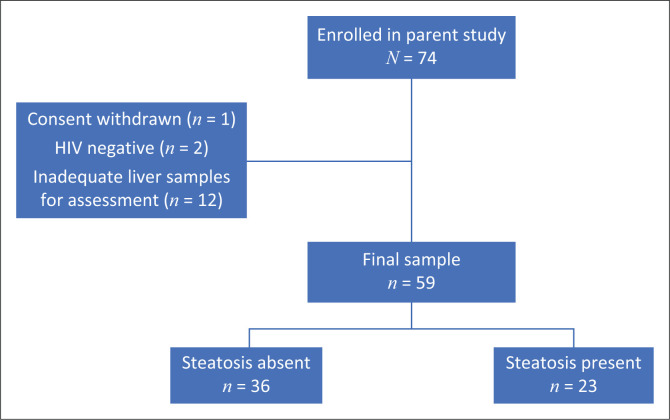
Flow diagram illustrating study enrolment process.

Of 74 decedents enrolled in the parent study, 59 met inclusion criteria for this study (Figure 1). The median age was 50 years (interquartile range [IQR]: 39–58); there were 33 male decedents (56%) and 26 female decedents (44%). Median CD4+ T-cell count was 139 cells/µL (IQR: 47–344) and 63% (37/59) were virologically supressed. Immunological discordance (virological suppression with CD4+ T-cell count < 200 cells/µL) was noted in 29% (*n* = 17/59).

### Histological review of liver minimally invasive tissue sampling

Steatosis was present in 39% (*n* = 23/59) of study decedents (Figure 1, Table 1). Of those with steatosis, 74% had mild steatosis, 9% moderate, and 17% severe. Macrovesicular steatosis was the dominant subtype at 91.3% (*n* = 21/23). One decedent was found to have both macrovesicular and microvesicular steatosis, and one had microvesicular steatosis only (Table 1). Of those with steatosis, there were none with associated portal or lobular inflammation to indicate the presence of steatohepatitis; one had mild fibrosis (Brunt score:1).

**TABLE 1:** Histological characteristics of minimally invasive tissue sampling biopsies in decedents with steatosis (*N* = 23).

Histological characteristics	*n*	%
**Steatosis severity**		
Mild: 5% to 33%	17	73.9
Moderate: 34% to 66%	2	8.7
Severe: > 66%	4	17.4
**Location**		
Peri-portal	4	17.4
Peri-venular	7	30.4
Diffuse	12	52.2
**Type of steatosis**		
Macrovesicular	21	91.3
Microvesicular	1	4.3
Both	1	4.3

The median age, in those with steatosis was 45.5 years (IQR: 39–56), and 51 years (IQR: 42–60.5) for those without (*P* = 0.2), and the median ART duration with steatosis was 47.5 months (IQR: 3.75–82.5), and 6 months without (IQR: 0–87) (*P* = 0.72) (Table 2). Duration on ART in those with and without steatosis categorised as ART naïve, < 6 months on ART, and > 6 months on ART showed no statistical significance (*P* = 0.79). There were no significant differences in liver enzyme tests between those who had steatosis and those who did not; however, alanine transaminase was lower in those with steatosis and approached statistical significance (Table 2).

**TABLE 2:** Clinical characteristics of decedents who underwent MITS stratified by steatosis being present or absent.

Characteristics	Steatosis present (*n* = 23)				Steatosis absent (*n* = 36)				Total (*N* = 59)	*P*
	Median	IQR	*n*	%	Median	IQR	*n*	%		
**Age at death (years)**	45.5	39.0–56.0	-	-	51	42.0–60.5	-	-	59	0.200
**Sex**	-	-	-	-	-	-	-	-	-	< 0.010*
Male	-	-	6	18.2	-	-	27	81.8	33	-
Female	-	-	17	65.4	-	-	9	34.6	26	-
**Body habitus (*n* = 44)†**	-	-	-	-	-	-	-	-	-	0.036*
Underweight	-	-	10	27.0	-	-	27	73.0	37	-
Normal/overweight	-	-	5	71.4	-	-	2	28.6	7	-
Median CD4-T-lymphocyte count	318	61.0–438.0	-	-	118	38.0–253.5	-	-	57	0.021*
**CD4-T-lymphocyte count in cells/μL**	-	-	-	-	-	-	-	-	-	0.030*
< 200	-	-	8	24.2	-	-	25	75.8	33	-
> 200	-	-	13	54.2	-	-	11	45.8	24	-
**Viral load**	-	-	-	-	-	-	-	-	-	0.790
Suppressed‡	-	-	15	40.5	-	-	22	59.5	37	-
Unsuppressed	-	-	8	36.4	-	-	14	63.6	22	-
**ART exposure**	-	-	-	-	-	-	-	-	-	0.530
Exposed	-	-	16	35.6	-	-	29	64.4	45	-
Unexposed	-	-	6	46.2	-	-	7	53.8	13	-
Median duration of ART in months	47.5	3.8–82.5	-	-	6	0.0–87.0	-	-	45	0.720
**Liver enzyme test median**										
Alanine transaminase	28	13.0–62.5	-	-	46	29.5–101.0	-	-	43	0.060
Aspartate transaminase	50	32.5–104.5	-	-	84.5	44.0–181.0	-	-	43	0.260
Alkaline phosphatase	129	81.5–646.0	-	-	156.5	83.5–217.0	-	-	43	0.690
Gamma-glutamyl transferase	50	33.0–145.5	-	-	96.5	48.0–243.0	-	-	41	0.280

IQR, interquartile range; ART, antiretroviral therapy.

* , Significance at *P* < 0.05.

† , Physician described;

‡ , Viral load < 200 RNA copies/mL.

### HIV-related risk factors for steatotic liver disease

A CD4 T-lymphocyte count of > 200 cells/µL was associated with an increased risk for steatosis (OR: 3.69; 95% CI: 1.19–11.44), while being virologically suppressed (viral load < 200 RNA copies/mL) was not (OR: 1.19; 95% CI: 0.40–3.54). Exposure to ART (OR: 0.64; 95% CI: 0.18–2.25) and to an ART regimen containing a protease inhibitor (OR: 0.48; 95% CI: 0.087–2.59) appeared to confer protection but did not reach significance, while duration on ART showed no association with steatosis (OR: 1.0; CI: 0.99–1.01). Decedents with a CD4 T-lymphocyte count > 200 cells/µL were also more likely to be normal/overweight (OR: 9.45; 95% CI: 0.95–94.48), another risk factor for steatosis.

### Non-HIV-related risk factors

**FIGURE 2:**
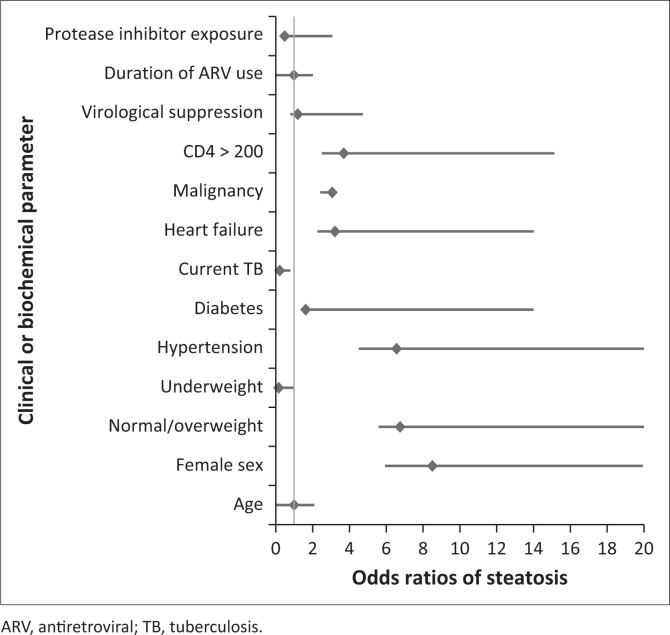
Odds ratios for risk of steatotic liver disease in virally suppressed and unsuppressed decedents with HIV. The point estimates and 95% confidence intervals are shown for each risk factor.

Female decedents (OR: 8.5; 95% CI: 2.57–28.17), being normal or overweight (OR: 6.75; 95% CI: 1.12–40.56), and having hypertension (OR: 6.56; CI: 2.05–21.00) were associated with an increased risk of hepatic steatosis (Table 3, Figure 2). Diabetes mellitus (OR: 1.62; 95% CI: 0.21–12.38), heart failure (OR: 3.21; 95% CI: 0.95–10.8), and having a malignancy (OR: 3.06; 95% CI: 0.65–14.29) also showed an increased risk of steatosis, but these did not reach statistical significance. Having active tuberculosis at time of death was associated with an 80% reduction in risk of steatosis (OR: 0.21; 95% CI: 0.06–0.58) (Table 3).

**TABLE 3:** Factors associated with hepatic steatosis in decedents who underwent minimally invasive tissue sampling.

Univariate analysis	Odds ratio	95% CI
Age	1.02	0.98–1.07
Female sex	8.50	2.57–28.17
Normal/overweight	6.75	1.12–40.56
Hypertension	6.56	2.05–21.00
Diabetes	1.62	0.21–12.38
Current tuberculosis	0.21	0.06–0.58
Heart failure	3.21	0.95–10.8
Malignancy	3.06	0.65–14.29
CD4 > 200 cells/μL	3.69	1.19–11.44
Virological suppression	1.19	0.40–3.54
Exposure to ART	0.64	0.18–2.25
Duration on ART	1.00	0.99–1.01
Exposure to protease inhibitor	0.48	0.09–2.59

ART, antiretroviral treatment; CI, confidence interval.

Serology for hepatitis B virus was available for 14 out of 59 (24%) decedents, six of whom had active disease; none of these six had steatosis. The data available do not allow for a finer evaluation of the association between steatosis and hepatitis B infection.

### Aetiology of steatosis

We were unable to attribute a cause for steatosis in 5 of the 23 decedents with hepatic steatosis; of the remaining 18, a single cause was attributed in 11, two causes in six, and three contributing causes in one case (26 causes) (Table 4). Criteria for MASLD were met in 9 out of 23 (39%) of decedents, representing 15% of the study population. Patients with a potential drug-related aetiology were often receiving antiretrovirals (ARVs) (tenofovir disoproxil fumarate, emtricitabine, and efavirenz), trimethoprim/sulfamethoxazole, and beta lactam antibiotics, or had previously been exposed to treatment for *Mycobacterium tuberculosis*.

**TABLE 4:** Likely aetiology of hepatic steatosis in decedents who underwent minimally invasive tissue sampling (*N* = 26).

Aetiology	*n*	%
Drug-related	8	30.8
HVOO	6	23.1
Alcohol	3	11.5
Obesity	3	11.5
Infection	2	7.7
Diabetes	2	7.7
Malignancy	1	3.8
Ischaemic	1	3.8

HVOO, hepatic venous outflow obstruction.

## Discussion

This study reports on the post-mortem prevalence of SLD in a cohort of people with HIV who died in hospital in the Matlosana district of North-West province, South Africa. We found a high proportion (39%) with hepatic steatosis, exceeding previously reported prevalences of 19.3% and 28% in living patients who had underlying liver disease from South African cohorts.^7,15^ It was also significantly higher than findings in PLWH from elsewhere in Africa, with reported prevalences of 3% in Zambia, using transient elastography, and 13% in Nigeria, using liver ultrasound.^5,16^ Our finding is also more than the estimated overall prevalence of steatosis in Africa of 13.5%.^4^ These differences may be the result of our study using liver biopsy, a more sensitive diagnostic modality than ultrasound and transient elastography.^17^ Other reasons may be the characteristics of our cohort of critically ill, hospitalised patients with multiple co-morbidities, including hypertension, cardiac failure, and a large proportion on ART. Our findings are similar to those reported in PLWH in the developed world, where prevalences range from 13% to 39.4%.^13,18,19^

While steatohepatitis is associated with greater liver dysfunction, simple steatosis is not benign. Steatosis causes functional impairment of hepatocytes, which may contribute to a diminished ability to respond to additional insults to the liver; this remains poorly elucidated. A recent meta-analysis reported that presence of steatosis is associated with risk for cardiovascular and metabolic disease, progression to non-alcoholic steatohepatitis, and with excess mortality compared to the general population.^20^ Our finding of immune reconstitution being a risk factor for steatosis is in line with others^6^; however, it differs significantly in severity, not being associated with MASH (41.7%) and fibrosis (21.7%), which were noted by others in studies where liver histology was available.^6^ In this study, steatosis was accompanied by relatively normal liver enzymes, supporting the absence of hepatocellular injury. There is increasing evidence that CD4+ T-lymphocytes drive inflammation and fibrosis in the liver,^21,22^ and may explain why our cohort with steatosis, but a low median CD4+ T-lymphocyte count of 318 cells/µL, had minimal inflammation and fibrosis compared to others where CD4 counts were higher, ranging between 377 cells/µL and 570 cells/µL.^6,19,23^ We found that virological suppression was not associated with risk for steatosis, as corroborated by others.^18,19,24^ The differences noted may be informed by our study population consisting of hospitalised, terminally ill individuals, with lower CD4 T-cell counts, rather than well-controlled outpatients.

We did not find that duration on ART was associated with risk for steatosis, which has been documented by others.^6,19^

### HIV-independent factors

In contrast to published data from high-income countries indicating higher prevalence of steatosis in men,^16,25,26^ we found steatosis predominantly in women, similar to previous findings in PLWH from the same hospital where biopsy-proven steatosis was more common in female patients than male patients (34% vs. 17%).^7^ Obesity was more prevalent in women in our study, consistent with findings that women in sub-Saharan Africa disproportionately suffer from hypertension and obesity.^27,28^ Women are also more likely than men to become overweight on a dolutegravir-containing regimen.^11^ These factors may explain regional variations in sex distribution of steatosis, and suggest a greater risk for MASLD in women.

Being normal/overweight was associated with significantly increased odds of having steatosis, in keeping with obesity being a well-established risk factor for SLD.^3^ Our study had a significantly higher proportion of underweight decedents (63%) than in comparable studies.^16,19,24^ Being underweight was associated with a 27% prevalence of SLD. While SLD has been described in lean and underweight individuals,^29,30,31^ we could not find studies describing SLD in underweight PLWH.

Hypertension, a risk factor for metabolic dysfunction, was a predictor of steatosis in our cohort. Patients with SLD are more likely to have uncontrolled hypertension and more cardiovascular events than hypertensives without SLD.^32^ Heart failure in this cohort tripled the odds of having steatosis, a finding approaching statistical significance. We found six cases with reasonable clinical and histological evidence that steatosis was secondary to venous outflow obstruction, in keeping with congestive hepatopathy and one case where hypotensive ischaemic hepatitis was considered. In both, ischaemic necrosis of hepatocytes impairs transport of triglycerides and results in accumulation of fat. In our cohort, no decedents had bridging fibrosis or cirrhosis, typically associated with prolonged haemodynamic instability. SLD, in isolation or as part of metabolic syndrome, is increasingly being recognised as a risk factor for cardiovascular disease.^33^

Diabetes mellitus, perhaps the best-described cause of MASLD, was a predictor for steatosis in our study but did not reach statistical significance, likely because of small sample size, and not all decedents being screened for the condition.

Malignancy was associated with a greater than three-fold increase in steatosis, but failed to reach statistical significance.

For those with established aetiologies for hepatic steatosis, we found 39% had more than one contributing cause, suggesting that causes of liver injury are often multifactorial and possibly cumulative in this population. Criteria for MASLD were met in 39% of those with hepatic steatosis, and 15% of the whole cohort, which may be an underrepresentation because markers of cardiometabolic risk were not requested ante-mortem for many participants. This difference in prevalences between our study and others may be confounded by the various definitions and diagnostic criteria associated with it that were previously used.

Severe steatosis (> 66% fat) was noted in 17% of our cohort. Worldwide, this ranges from 1% to 27.3% in PLWH,^16,19,24^ and reflects the diverse baseline characteristics in genetics, lifestyle, and management of HIV.

### Limitations

Our small sample size precluded a multivariate analysis of data and a more accurate demonstration of association. Missing data may have impacted outcomes; in particular, alcohol use quantification, nutritional status, and data regarding metabolic risk stratification used in the diagnosis of MASLD. Hepatitis B and C virus co-infection, both drivers of SLD and steatohepatitis, could not reliably be assessed as these tests were not routinely requested ante-mortem. This single study site, and the use of critically ill hospitalised decedents may have impacted our findings.

## Conclusion

This study found a high prevalence of steatosis of multiple and overlapping aetiologies in terminally ill PLWH. In addition to well-known risk factors, we found female sex and a preserved CD4 count were significant risk factors for SLD, findings not previously described. In addition, more than a quarter of underweight PLWH had steatosis, indicating a need to better characterise the pathogenesis of steatosis in this subgroup. This study has identified a subgroup of PLWH who may be at higher than previously appreciated risk for SLD and who may benefit from closer clinical follow-up and management of risk factors. Our findings underscore a need for more research to delineate the nuanced interplay of HIV infection, ART, and risk factors for development of hepatic steatosis, with focus on the sub-Saharan patient, to inform tailored interventions for patient care in this population.
